# Healthy Eating Is Associated with Sarcopenia Risk in Physically Active Older Adults

**DOI:** 10.3390/nu13082813

**Published:** 2021-08-16

**Authors:** Konstantinos-Georgios Papaioannou, Andreas Nilsson, Lena Maria Nilsson, Fawzi Kadi

**Affiliations:** 1 School of Health Sciences, Örebro University, 702 81 Örebro, Sweden; konstantinos.papaioannou@oru.se (K.-G.P.); fawzi.kadi@oru.se (F.K.); 2 Epidemiology and Global Health, Umeå University, 901 87 Umeå, Sweden; lena.nilsson@umu.se

**Keywords:** muscle mass, aging, physical function, handgrip strength, Healthy Diet Score (HDS), dietary patterns, physical activity, sarcopenia risk, diet quality, muscle strengthening activities

## Abstract

Healthy Diet and physical activity may play important roles in the maintenance of muscle health during aging. The aim of the present study was to explore the impact of adherence to healthy dietary patterns on sarcopenia risk in a sample of physically active older men and women, while considering adherence to guidelines on muscle strengthening activities (MSA) and protein intake. Based on a sample of 191 physically active men and women (65–70 years), dietary intake was assessed using a 90-items food-frequency-questionnaire (FFQ) and Healthy Diet Score (HDS) was calculated. Physical activity was assessed by accelerometry and self-report. A sarcopenia risk score (SRS) was derived based on three indicators of muscle health: muscle mass was assessed using bioelectrical impedance and handgrip strength and 5 times sit-to-stand (5-STS) were determined by standardized procedures. Analysis of covariance (ANCOVA) was used to examine differences in SRS and its components across sex-specific tertiles of HDS, with adjustments for covariates including total energy intake, protein intake and MSA. A significant main effect (*p* < 0.05) of HDS on SRS was observed, where those belonging to the highest HDS tertile had lower SRS compared to those in the lowest tertile. A corresponding significant effect was observed for 5-STS performance, with better performance in those with the highest HDS adherence compared to those with the lowest. The present study supports guidelines emphasizing diet quality beyond amounts of macro- and micronutrients in the prevention of age-related deterioration of muscle health. Importantly, the benefits from healthy dietary patterns are evident in older adults who already adhere to guidelines for health-enhancing physical activity.

## 1. Introduction

Aging is accompanied by a progressive decline in skeletal muscle mass and function, which ultimately leads to an increased risk of developing sarcopenia [1], defined as a generalized skeletal muscle disorder characterized by an increased risk of falls, physical disability and mortality [1,2]. A clinical definition of sarcopenia based on the three components; muscle strength, muscle mass and measures of physical performance, has been issued by the European Working Group on Sarcopenia in Older People 2 (EWGSOP2) [1] In this respect, lifestyle behaviors, including diet and physical activity may play an important role in preventing the occurrence of sarcopenia [3,4,5]. Indeed, several reports have highlighted the putative role of proteins on muscle health [6], where adherence to a recommended daily intake of 0.8 g·kg^−1^ bodyweight (BW) has a beneficial impact on muscle mass [7], whereas a consumption greater than 1.1 g·kg per day is linked to both higher muscle mass and muscle function in older adults [8,9]. The exploration of the impact of overall dietary patterns, rather than single nutrients, on skeletal muscle health has the advantage of considering the complex interplay and potential synergistic effects by different foods on health outcomes. In this respect, a series of studies suggested that healthy dietary patterns, characterized by greater fruit, vegetable and wholegrain consumption, may infer beneficial effects on muscle strength and function [10]. However, less evidence is available in older adults and previous studies often considered isolated parameters of muscle function, which unlikely covers overall dimensions of muscle health [10]. Moreover, a recent systematic review reported inconsistencies in the relationship between dietary patterns and indicators of muscle health, which highlights the need for further studies investigating the role of dietary habits diet on muscle health in older adults [11]. An important step towards the understanding of the independent influence of diet on determinants of sarcopenia risk is to consider, in general, the potential myotrophic effects of physical activity (PA) habits and, more specifically, muscle-strengthening activities (MSA) [12,13]. Indeed, major health organizations, including WHO, highlight the beneficial role of MSA in promotion of muscle health in older adults [14]. Several biological factors may mediate the putative effects of dietary patterns on muscle health. For example, detrimental effects of a pro-inflammatory environment on muscle health have previously been documented [15]. Furthermore, there is an interplay between elevated inflammatory status and abdominal obesity [16], which in turn may accelerate the rate of muscle wasting by advancing age. Therefore, such biological factors need to be considered when elucidating links between healthy eating and indicators of muscle health in older adults.

The aim of the present study was to explore the impact of overall dietary patterns on components of the sarcopenia risk in a sample of physically active older men and women.

## 2. Materials and Methods

### 2.1. Participants

A total of 252 community-dwelling Swedish older adults (men and women) aged 65–70 years old were recruited through local advertisement during 2018. Exclusion criteria were: presence of overt disease including diabetes mellitus, coronary heart disease, musculoskeletal disorders, psychiatric disease and mobility disabilities. In addition, participants who accumulated less than 150 min/week of moderate-to-vigorous physical activity (MVPA) were excluded. A written informed consent was obtained from participants and all investigations were performed in accordance with the Declaration of Helsinki ethical principles. The Regional Ethics Committee of Uppsala, Sweden, approved the study (Dnr 2017/511).

### 2.2. Assessment of Dietary Intake

Dietary data were collected using a 90-items food-frequency questionnaire (FFQ) based on a validated 84- and 65-items FFQ, developed within the Västerbotten Intervention Programme (VIP) [17,18]. All participants received instructions face-to-face by a single investigator prior to filling out the FFQ. The FFQ has 9 fixed alternatives (never, occasionally, 1–3 times/month, 1 time/week, 2–3 times/week, 4–6 times/week, 1 time/day, 2–3 times/day, ≥4 times/day) for assessing the intake frequency. The Healthy Diet Score (HDS), was derived on the basis of reported intakes of favorable (fish, fruits (except juices), vegetables (except potatoes) and whole grains) and unfavorable (red or processed meats, desserts and sweets, sugar-sweetened beverages and fried potatoes) food groups [19]. The frequency intake of each food group was ranked in sex-specific quartiles and assigned an ascending (0, 1, 2, 3) or descending (3, 2, 1, 0) value, for favorable and unfavorable food groups, respectively. The quartile ranks were summed into the Healthy Diet Score (min 0; max 24), where a higher rank indicates healthier dietary pattern. Based on HDS, participants were divided into sex-specific tertiles reflecting adherence to a healthy diet (Low, Moderate and High). In addition, total energy intake and daily protein intake were retrieved. The protein cut-point of 1.1 g·kg^−1^ BW was used to determine adherence to guidelines on protein intake in older adults [20].

### 2.3. Assessment of Anthropometry and Components of Sarcopenia Risk 

Standard anthropometrical procedures were used to assess body height and weight. Waist circumference (WC) was measured at the midpoint between the lower costal margin and the iliac crest using measuring tape. Bioelectrical impedance analysis (Tanita MC-780, Tanita Amsterdam, The Netherlands) was used to assess skeletal muscle mass index (SMI). Skeletal muscle mass (SMM) was computed using the Janssen equation [21], and expressed as a percentage of body weight (SMI, %). Indices of muscle function were assessed as follows: handgrip strength (HG) using a Jamar hand dynamometer (Patterson Medical, Warrenville, IL, USA) and the five times sit-to-stand test (5STS), where participants were instructed to sit down in a chair, starting from a standing upright position and to repeat this sequence 5 times. Based on current operational definition issued by the EWGSOP2 [1] sarcopenia is clinically diagnosed based on an integration of the following components: low muscle strength and low muscle mass, and low physical performance as an indicator of severity. Therefore, in line with this definition and in accordance with a large body of previous research, we aggregated a standardized sex-specific score denoting sarcopenia risk according to the following: firstly, the three variables were standardized (z-scores) in men and women separately. Secondly, the three sex-specific standardized variables were averaged into one single SRS variable. Importantly, the SRS cannot classify whether individuals have sarcopenia, but rather denotes higher or lower risk of sarcopenia based on an aggregation of sarcopenia risk components as used in previous work [22]. 

### 2.4. Assessment of Adherence to PA Guidelines

The Actigraph GT3x (Actigraph, Pensacola, FL, USA) accelerometer was used to assess adherence to the PA guideline regarding 150 weekly min of MVPA, as previously described [23]. Briefly, participants were instructed to wear the monitor around the waist during all waking hours (except water-based activities) for a period of seven days. A minimum of four days with at least 10 h of wear time per day was required for inclusion. Non-wear time was defined as a minimum of 60 min of continuous zero accelerometer counts. Time spent in MVPA was determined based on the established accelerometer count cut-point of >2019 counts per minute [24]. Participants accumulating an average of 22 min of MVPA per day (approximating 150 min per week) were classified as meeting current guidelines about health-enhancing PA issued by the WHO [14]. Adherence to MSA was assessed using the EPAQ2 questionnaire [25] where participants reported on duration and frequency of MSA during the last 12 months. Participants were then stratified into two groups (≥2 sessions per week or less).

### 2.5. Assessment of High-Sensitivity C-Reactive Protein (hs-CRP)

Blood samples were collected from an antecubital vein after an overnight fast. Subjects were asked not to engage in any strenuous physical activity and avoid smoking and alcohol 24 h before the sampling. An automated immunoturbidimetric assay (Advia 1800, Chemistry System, Siemens, Germany) using a Hs-CRP kit was employed to assess Serum CRP level.

### 2.6. Statistical Analysis

Data are presented as means ± SD, unless otherwise stated. Normal distribution was checked by visual inspection and Shapiro-Wilks tests and data was log-transformed when necessary, to fit a normal distribution. Sex-differences in general characteristics were assessed using independent samples T-test. Analysis of covariance (ANCOVA) was used in order to determine the effects on components of SRS across tertiles of HDS. First, analysis was adjusted by total energy intake and thereafter the adherence to guidelines for protein intake (yes/no) and to the MSA guideline (yes/no) were added as fixed factors, with waist circumference and level of CRP as continuous covariates. In addition, post-hoc tests with Bonferroni correction were performed to examine the differences between tertiles. A priori power calculation indicated the detection of small to moderate effect sizes on SRS with a power of ≥80% when based on the study sample size and an alpha level of 0.05. All statistical analyses were performed using SPSS Statistics, version 27.0 (IBM Corp., Armonk, NY, USA).

## 3. Results

The final analyses included a total of 69 men (67.4 ± 1.5 years) and 122 women (67.4 ± 1.6 years) who accumulated 150 min/week of MVPA and had complete data on all variables. Significant sex-specific differences were observed for all anthropometric variables and HG, whereas no such difference was observed for 5STS performance (Table 1). 

There were no sex-differences in CRP levels (men: 1.0 ± 2.6 mg/L vs. women: 1.0 ± 2.5 mg/L). Total energy intake was significantly higher in men (2146 ± 612 kcal) compared to women (1540 ± 472 kcal), whereas no corresponding differences were observed in relative protein intake (men: 1.01 ± 0.32 g·kg^−1^ BW vs. women: 0.99 ± 0.31 g·kg^−1^ BW). Median intakes of the eight HDS food items are presented stratified by tertiles of HDS adherence in Table 2. 

**Figure 1:**
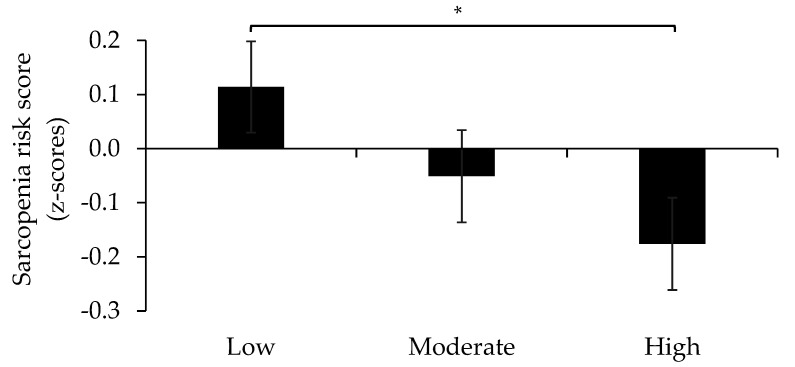
Sarcopenia risk score (SRS) across tertiles of the Healthy Diet Score (HDS). * *p* < 0.05.

To examine the effect of HDS on components of SRS sarcopenia risk, we first analyzed differences across tertiles of HDS adherence, while controlling for total energy intake. Our analysis revealed a significant main effect (*p* < 0.05) of HDS on SRS. We further adjusted the model by adherence to protein and MSA guidelines as well as waist circumference and CRP levels, which left the beneficial impact of HDS on SRS significant (*p* < 0.05). Post-hoc analyses revealed a significantly lower SRS in those with highest HDS adherence compared to those with the lowest adherence (Figure 1). 

**Figure 2:**
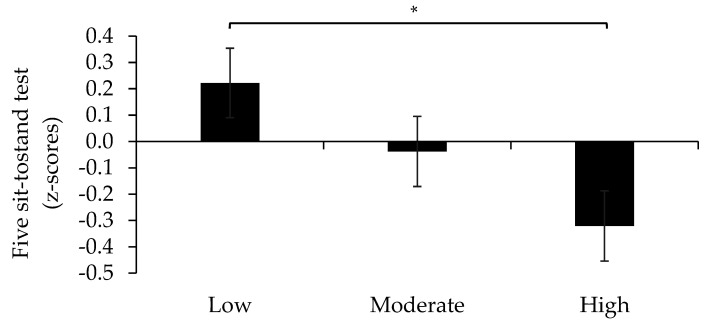
Five sit-to-stand test (5STS) performance across tertiles of the Healthy Diet Score (HDS). A higher score denotes lower 5-STS performance. * *p* < 0.05.

**Figure 3:**
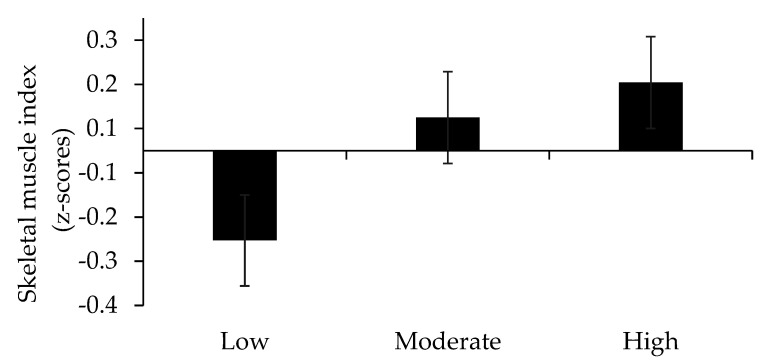
Skeletal muscle mass index (SMI) across tertiles of the Healthy Diet Score (HDS). A lower score denotes lower SMI.

**Figure 4:**
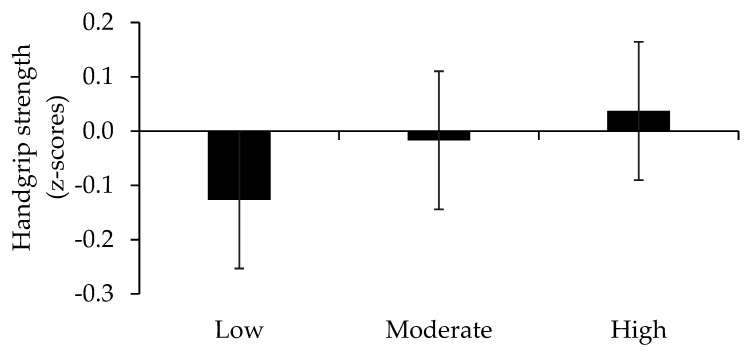
Handgrip strength (HG) across tertiles of the Healthy Diet Score (HDS). A lower score denotes lower HG.

We further explored the impact of HDS on each single component of SRS and found significant differences in 5STS across HDS tertiles in covariate adjusted models, where those with highest HDS adherence had a better 5STS (*p* < 0.05) performance compared to those with the lowest adherence (Figure 2). Although main effects of HDS on SMI and HG were non-significant, similar trends to that observed for 5STS were indicated (Figure 3 and Figure 4).

## 4. Discussion

The present study highlights the beneficial link between healthy eating and sarcopenia risk in physically active older men and women. Importantly, this finding was evident even when the engagement in muscle strengthening activities and adherence to guidelines for protein intake were taken into consideration, suggesting an important role of diet quality as an independent factor able to mitigate sarcopenia risk in older adults.

Our study extends previous work on the role of overall diet quality on muscle health [11,26,27,28,29,30] in a sample of physically older men and women, where all participants adhered to the guideline about weekly MVPA time. Given that reduced muscle activity decreases muscle mass in older adults [31] and that engagement in muscle strengthening activities lowers sarcopenia risk in older adults [13], our findings suggest that adherence to a healthy diet has beneficial effects on sarcopenia risk beyond those inferred by adherence to guidelines regarding MVPA time and engagement in muscle strengthening activities. Moreover, the observed link between HDS and SRS was independent of both total macronutrient intake and adherence to guidelines for protein intake, which suggests that dietary quality can elicit beneficial impacts on the preservation of muscle health that are separate from those driven by adequate amounts of daily protein intake. Indeed, adequate protein intake is an established core dietary element linked to muscle mass and function [22,32] and an intake of at least 1.1 g·kg^−1^ BW infers beneficial effects on muscle mass and muscle function in older adults [9]. Therefore, in addition to the importance of protein intake, particular attention should be paid to the overall diet quality when conceptualizing nutritional approaches aiming to mitigate sarcopenia risk in older adults.

Several presumed mechanisms have been put forward to account for the beneficial effects of healthy diets on skeletal muscle. It is currently hypothesized that the overall health benefits of healthy eating are partly due to the modulation of the systemic inflammatory environment [33]. In this respect, serum level of CRP is an established marker of systemic inflammation and is recognized as an important factor underlying several age-related deleterious changes including muscle wasting [15]. However, a notable finding in our study was that variations in CRP levels did not affect the proposed effects of adherence to a healthy diet on sarcopenia risk. Thus, our findings indicate that the observed links between dietary patterns and SRS are not influenced by variations in CRP levels. However, our sample of physically active older men and women are likely to benefit from the MVPA-related attenuation of systemic inflammation as indicated in a previous work [34], which may mask the involvement of the inflammatory environment on links between dietary patterns and muscle health. Further investigations, preferably encompassing a wider set of inflammatory biomarkers, are warranted in order to elucidate the complex interplay between the inflammatory environment, muscle health and lifestyle behaviors. It is also hypothesized that healthy diets rich in fruit and vegetable may protect against metabolic acidosis and reduce proteolysis and amino acid catabolism [35], thus mitigating sarcopenia risk [36]. Additionally, an unfavorable dietary pattern, including foods rich in saturated fats may be detrimental for maintenance of muscle health [37], whereas diets rich in fiber may mitigate sarcopenia risk [38]. Altogether, the impact of dietary patterns on muscle function of older adults are unlikely to be explained by the action of single nutrients or single biological pathways. Further research is warranted in order to understand how healthy dietary patterns influence age-related decline in muscle health.

Our findings also indicate that impacts of HDS on muscle health were identified when assessing an indicator of lower muscle strength and performance but not skeletal muscle mass. Importantly, it may be hypothesized that any myotrophic effects of healthy eating are less likely to be detected in our sample of older adults, who already adhere to the general guidelines of time in MVPA, compared to less active groups of older adults. However, although not significant, trends indicating beneficial impacts of adherence to HDS on SMI and HG could, nevertheless, be observed, which warrants further investigations, preferably based on larger samples. 

The strength of the present study is the adjustment of findings by important variables with the potential to influence on components of sarcopenia risk, which contributes to the robustness of the findings. The VIP FFQ has been validated both in its long 84 food item version [17], and in the short 64 food-item version which includes merged categories from the 84 food item version, at least with regards to the intake of folate and vitamin B [18]. It is generally agreed that the validity of FFQs increases by the number of food items included [39]. Thus, there is no reason to believe that the validity of our FFQ would be negatively affected by adding 6 extra food items. Indeed, the exploration of the impacts of HDS has been performed while considering both the influences of adherence to the guidelines for protein intake and engagement in muscle strengthening activities, both well-established factors contributing to muscle health. However, the study is not without limitations. For example, directions of associations cannot be determined given the cross-sectional design. Further, in order to address the question of whether healthy diet has an impact on SRS beyond those inferred by health-enhancing time spent in MVPA, the selected sample of older adults is unlikely to be representative of a larger sample of older adults where there is a greater variation of ethnicity, socio-demographic backgrounds, and health status. Furthermore, although several important covariates have been considered, residual confounding may still exist. Finally, despite the highest accuracy of computed tomography and magnetic resonance imaging, skeletal muscle mass was assessment by the use of bioelectrical impedance (BIA) analysis, which is more feasible for larger-scale studies. Noteworthy, in standardized conditions and using previously cross-validated equations, the BIA method is currently considered as a valid tool for measurement of functioning muscle mass in clinical settings as also acknowledged by the European Working Group on Sarcopenia in Older People 2 (EWGSOP2) [1].

## 5. Conclusions

In conclusion, the present study highlights the impact of healthy eating on components of sarcopenia risk in physically active community-dwelling older adults. This finding supports guidelines emphasizing diet quality beyond amounts of macro- and micronutrients in the prevention of age-related deterioration of muscle health. Our study also supports the promotion of healthy eating in older adults who already adhere to guidelines for health-enhancing physical activity.

## Figures and Tables

**Table 1:** Anthropometrics and components of the sarcopenia risk score (SRS).

	Men	Women
**Anthropometrics**		
Height (cm)	178.2 ± 6	164.6 ± 5.8 *
Weight (kg)	79.9 ± 11.1	63.4 ± 9.0 *
BMI (kg/m^2^)	25.1 ± 3.0	23.4 ± 3.2 *
Waist circumference (cm)	93.4 ± 9.9	78.9 ± 8.5 *
**Sarcopenia Risk**		
Skeletal Mass Index (%)	34.6± 3.1	26.9 ± 3.4 *
Hand grip per body weight (g·kg^−1^ bodyweight)	0.56 ± 0.10	0.45 ± 0.08 *
5 Sit-to-Stand test (s)	10.1 ± 1.9	10.2 ± 2.3

Data are expressed as mean ± SD. * *p* < 0.05 between sexes.

**Table 2:** Intakes (g/day) of the eight components of the Healthy Diet Score (HDS) stratified by tertiles of HDS.

	**Low HDS (*n* = 69)**	**Moderate HDS (*n* = 61)**	**High HDS (*n* = 61)**
**Favorable food groups**			
Vegetables	81.5 (36.7–148.5)	125.2 (75.8–200.6)	176.6 (113.4–247)
Fruit	110.6 (70.6–196.4)	165.4 (90.3–239.7)	236.8 (161.9–317.2)
Fish	24.8 (20.3–32.7)	29.5 (18.1–45.0)	35.9 (24–46.3)
Whole grain	55.9 (35.1–68.0)	57.8 (38.4–78.4)	67.4 (50–91.4)
**Unfavorable food groups**			
Sugar sweetened beverages	34.5 (12.7–107.1)	19.1 (1.3–89.7)	12.6 (1–54.3)
Red/processed meat	55.1 (41.8–74.7)	48.5 (29.7–62.9)	36.1 (13.4–54.8)
Desserts and sweets	14.4 (9.6–28.7)	11.1 (7.4–23.0)	9.7 (5.0 –13.1)
Fried potatoes	14.0 (8.0 –21.4)	8.2 (0.5–17.1)	8.2 (0.53–13.7)

Data are expressed as median (IQR).

## Data Availability

Data supporting reported results are available upon reasonable request and in accordance to the ethical principles.
